# Study on the Modification of Silty Soil Sites Using Nanosilica and Methylsilicate

**DOI:** 10.3390/ma16165646

**Published:** 2023-08-16

**Authors:** Pengke Cao, Qingwen Ma, Mingming Zha, Jian Zhang, Zijian Huo

**Affiliations:** School of Water Conservancy and Transportation, Zhengzhou University, Zhengzhou 450001, China

**Keywords:** silty soil site, nano silica, potassium methylsilicate, modification test, mechanism of action

## Abstract

The special particle grading properties of silt lead to the strong water sensitivity and low soil strength of silt sites, many of which are severely damaged and urgently need to be repaired. This article takes the powder soil from a certain burial site area in Xizhu Village, Luoyang as the research object, which is improved by adding nanosilica and potassium methylsilicate. The modified soil is studied through mechanical and waterproof performance tests, and the mechanism of action of the modified material is analyzed through SEM and XRD. The experimental results show that the mechanical properties and waterproof properties of the composite modified soil were improved when the nanosilica content was 2% and the potassium methylsilicate content was 0.5%; the durability of the composite modified soil is improved, making this the optimum ratio. The mechanical properties and water resistance of the silty soil were significantly improved by adding the appropriate amount of nanosilica and potassium methylsilicate. Nanosilica can be evenly dispersed in the soil matrix, absorb a small amount of water to form a gel state, fill the pores in the silt aggregates, and improve soil compactness. In addition, nanosilica aggregates can attach to the surface of the soil particles and extend from the particle surface to the particle edge. By increasing the contact between soil particles and increasing the particle size, the mechanical properties of the modified soil are improved. When potassium methylsilicate solution is added to the soil, it reacts with water and carbon dioxide, decomposes into methylsilicate, and quickly generates a polymethylsiloxane film to cover the surface of soil particles, forming a waterproof film on the surface and thereby improving the waterproof performance of modified soil. Our research results can provide a reference for the restoration and protection of silty and silt-like sites. The next step is to apply the composite modified soil in engineering restoration through field tests in order to study the repairing ability of composite modified soil and its actual protective effects.

## 1. Introduction

China is a country with a long cultural history, with much precious cultural heritage. As an important part of this heritage, earthen ruins have witnessed the rich and colorful history and culture of the Chinese nation and are one of our precious cultural treasures [1]. Because most such sites have been exposed to the natural environment for a long time, they are often affected by natural factors such as wind, sun and rain and suffer from multiple sources of damage, including the dry–wet cycle, freeze–thaw cycle, and biological erosion. Relevant studies have begun to examine how to repair earthen sites as a means to protect this important cultural heritage. At present, the most commonly used methods include chemical reinforcement, crack grouting, and anchoring technology [2], which aim to enhance the structural stability and strength of the site to cope with the threat of changes in the natural environment and man-made destruction. It should be noted that all of the above methods have a certain impact on earthen ruins. Following the principle of “minimum intervention” in the protection of cultural relics [3], in this paper we plan to use modified materials to improve the site soil and to apply modified soil for the protection and restoration of original sites to improve their waterproof and mechanical properties. This method is a very effective protection measure, and can protect heritage sites to the greatest extent.

Beginning in 1995, Li Zuixiong and others [4,5] have used PS materials to strengthen earthen ruins, and have found that these kinds of materials can improve their water resistance and mechanical properties. In addition, after many experimental studies it has been found that PS, RTV, WD-S, and other reinforcement materials can effectively improve the mechanical and waterproof properties of soil [6,7,8]. In 2014, Bahmani et al. [9] studied the effects of silicon dioxide nanoparticles on the consistency, compaction, hydraulic conductivity, and compressive strength of cemented soils. In addition, it has been found that the pozzolanic reaction of cement can be promoted by micro-experiment. In 2015, Changizi et al. [10] found that the application of nanosilica and recycled polyester fiber can significantly improve the shear strength, unconfined compressive strength, and elastic modulus of clay in soft clay improvement. Their research results show that nanosilica plays a more significant role in improving unconfined compressive strength than recycled polyester fiber. In 2016, Ren Xiaochuan and others [11] conducted microscopic tests and found that nanosilica improved the uniformity of pores in soil sensitive to frost heaving, thereby improving the mechanical properties of soil. In the same year, Ghasabkolaei and others [12] verified through experiments that adding nanosilica to cement-improved clay can effectively improve its unconfined compressive strength. These research results provide a useful reference for the application of nanosilica in the field of soil improvement. In 2017, Qu Jili et al. [13] found that the joint application of basalt fiber and nanosilica can improve the shear strength and cohesion of clay, though it had little effect on improving the internal friction angle. Moreover, it had a more significant effect on improving the mechanical properties of soil after determining the optimal content. In 2018, Shang et al. [14] explored the effects of adding nano-alumina and nanosilicon dioxide to expansive soils; their research showed that the best compression index was obtained by adding 1.2% nano-alumina and 2% nanosilicon dioxide into expansive soil; on the other hand, the unconfined compressive strength of expansive soil was maximized by adding 1.2% nano-alumina and 1.5% nanosilicon dioxide. In the same year, Lv et al. [15] studied the effect of nanosilica with different sizes in loess solidification, and found that the mechanical strength of loess decreased with increasing nanoparticle size. In 2020, Tomar et al. [16] studied the effect of clay modified by nanosilica and polypropylene fiber; their research found that the compressive strength of clay increased with increasing content of composite modified materials. In 2021, Tian Zhenkun and others [17] modified silica sol with potassium methylsilicate and explored the influence of different mass fractions of potassium methylsilicate on silica sol coatings. Their results showed that potassium methylsilicate effectively improved the hydrophobicity and water resistance of the coatings. When 5% potassium methylsilicate was added, the water absorption was only 0.9% and the coating performance was good. In 2022, Lv et al. [18] studied the hydrophobic modification effect of composite potassium methylsilicate on the gel skeleton of alkali slag finding that a hydrophobic group (-CH_3_) could be grafted onto the chemical structure of C-A-S-H/C-S-H gel through condensation reaction.

In their research, the above researchers found that nanosilica is a material that can significantly improve the strength of soil. However, at present the relevant research is mainly focused on clay and loess, and research on silty sites is relatively rare. After modification of silica sol by potassium methylsilicate (PMS), the hydrophobicity and water resistance of silica sol coatings can be significantly improved; however, applications of potassium methylsilicate for the protection of soil are relatively few. Therefore, in this paper we explore the application of nanosilica and potassium methylsilicate for soil protection of silty sites.

The silty soil of a tomb site in Xizhucun, Luoyang, was modified by adding nanosilicon and potassium methylsilicate. The optimal content of nanosilica and potassium methylsilicate was determined by mechanical property tests and waterproof performance tests, and were found to be 2% and 0.5%, respectively. The mechanism of soil co-modification by nanosilicon and potassium metasilicate was studied by SEM and XRD. The best ratio of modified soil will be used to repair the site, providing a reference for the restoration and protection of silty soil sites.

## 2. Experiment Scheme

### 2.1. Experimental Materials

The test materials included silt, nanosilica and potassium methylsilicate. The soil was crushed, air-dried, and passed through a 2 mm sieve. The basic physical properties of the soil samples were determined according to ASTM D4318 [19], as shown in Table 1.

The test results of soil particle analysis by sieving and densimeter are shown in Table 2.

The specific parameters of the nanosilica (NS) are shown in Table 3. Potassium methylsilicate (PMS) is a new type of rigid silicone waterproof material which has been widely used in waterproof engineering of various building materials, such as stone, ceramics, cement mortar, perlite, etc. This material has appropriate strength and good compatibility with the matrix to be repaired; at the same time, it causes less pollution to the environment during its production and use.

### 2.2. Experimental Method

**(1) Direct Shear Testing.** When making samples, the plain soil is naturally dried first, then the plain soil is screened and the particle size is controlled within 2 mm. According to the geotechnical test code (GB/T50123-2019) [20], the sample is a circular knife sample with a diameter of 60 mm and a height of 20 mm, and the water content is controlled at about 12.6% (optimal water content). Shear tests were carried out under vertical pressures of 100, 200, 300, and 400 kPa, respectively, and the shear rate was 0.8~1.2 mm/min.

**(2) Capillary Water Rise Testing.** When making samples, the plain soil is first naturally dried, then the plain soil is screened and the particle size is controlled within 2 mm. The sample is a cylindrical sample with a diameter of 39.1 mm and a height of 80 mm, and the water content is controlled at about 12.6% (optimal water content). The permeable stone is placed in a glass water tank and the sample is placed on the permeable stone; the water level will exceed the bottom surface of the permeable stone by 2–3 mm, while the water level will not exceed the top surface of the permeable stone. The rising height of capillary water is marked every 5 mm, and the rising of water is recorded as it passes through the sample until the water reaches the top of the sample surface [21,22].

**(3) Scanning Electron Microscopy (SEM) Analysis.** The experimental instrument used to measure and analyze the microscopic appearance of plain soil and soil modified by nanosilica and potassium methylsilicate was a Quanta 650 environmental scanning electron microscope produced by American FEI.Founded in 1971 in the United States, FEI is a leading manufacturer and operator of a wide range of scientific instruments, providing world-class microscopy solutions to the global nanotechnology community.

**(4) X-Ray Diffraction (XRD) Analysis.** The experimental instrument was a Brooke D8 X-Ray diffractometer made in Saarbrücken, Germany, and the scanning angle was 0~80. The phase compositions of plain soil and soil modified by nanosilica and potassium methylsilicate were analyzed.

## 3. Results and Discussion

### 3.1. Modification Experiment of Single Doping by Nanosilica

#### 3.1.1. Direct Shear Testing

**Figure 1:**
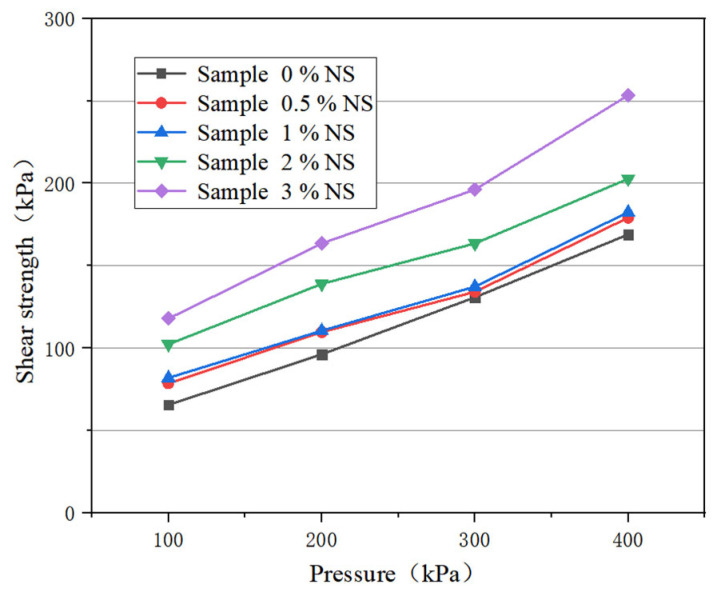
Shear strength curve.

According to the different dry mass ratios of nanosilica to dry powder soil, the tested proportions of nanosilica were 0, 0.5%, 1%, 2%, and 3%, respectively. The shear strength results of the sample are plotted in Figure 1. It can be seen that the shear strength of the samples with nanosilica are improved compared to the sample without nanosilica. Under the same vertical pressure, the shear strength of the samples increases with the increase in the nanosilica doping ratio. Under vertical pressure of 100 kPa, the shear strength of the samples with 0.5%, 1%, 2%, and 3% nanosilica content is 78.6 kPa, 81.9 kPa, 102.3 kPa, and 117.9 kPa, respectively, which is 13.1 kPa, 16.4 kPa, and 36.8 kPa higher, respectively, than the sample without nanosilica.

The main reason for the increase in shear strength is that nanosilica is added to silt mainly by filling pores [23,24,25], which increases the compactness of soil, resulting in the shear strength of the sample being significantly increased on the macroscopic level.

**Figure 2:**
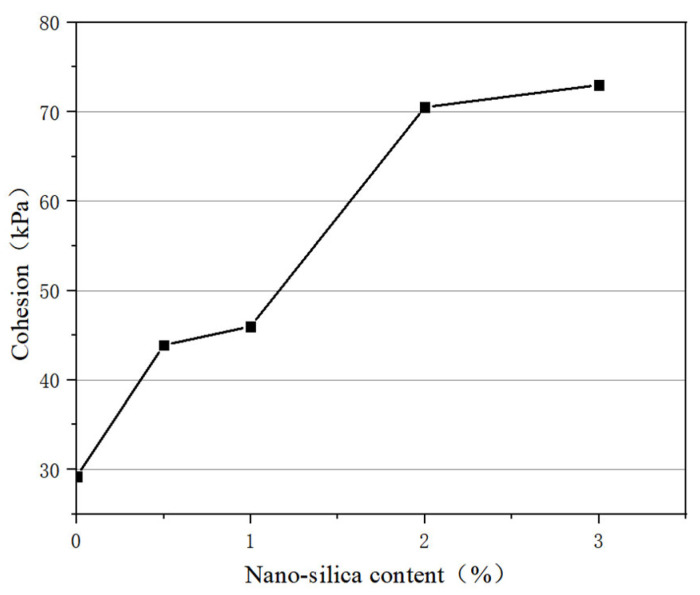
Variation of cohesion with nanosilica content.

According to the Moore–Coulomb theory, the intercept of the shear strength fitting curve represents the cohesion of the soil and the slope represents its internal friction angle. The shear strength curve was fitted according to the results of the direct shear test to obtain the cohesion and internal friction angle of the soil, then the graph of the cohesion and nanosilica content was drawn, as shown in Figure 2. The cohesion is mainly the stress caused by the mutual attraction between soil particles. It can be seen that the cohesion increases with the increase in nanosilica content. Among the samples, the cohesive force of the modified soil with 3% nanosilica is the largest, reaching 73 kPa, which is 43.8 kPa higher than the value of 29.2 kPa without nanosilica. When the content of nanosilica is 0.5%, the cohesion reaches 43.9 kPa, which is 14.7 kPa higher than without nanosilica. When the content of nanosilica is 1%, the cohesion reaches 46 kPa, which is 16.8 kPa higher than without nanosilica. When the content of nanosilica is 2%, the cohesion reaches 70.5 kPa, which is 41.3 kPa higher than without nanosilica.

**Figure 3:**
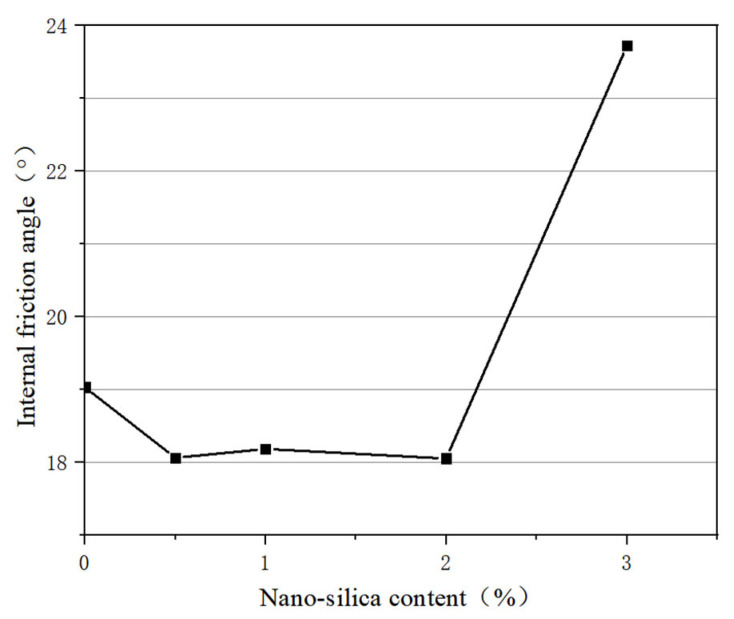
Variation of internal friction angle with nanosilica content.

The graph of the internal friction angle and nanosilica content is shown in Figure 3. The internal friction angle reflects the friction characteristics caused by the interaction between soil particles. It can be seen that the change of internal friction angle with nanosilica content has no obvious regular characteristics.

In summary, the shear strength and cohesion of silt increase with the increase in nanosilica content. When the nanosilica content is 2%, the increase in cohesion is quite different than with 1% nanosilica, while there is little difference between the increase in cohesion with 3% and 2% nanosilica. Moreover, a higher content of nanosilica can aggravate the brittleness of soil, leading to increased project costs, and the internal friction angle does not change with the content of nanosilica.

#### 3.1.2. Capillary Water Rise Testing

**Figure 4:**
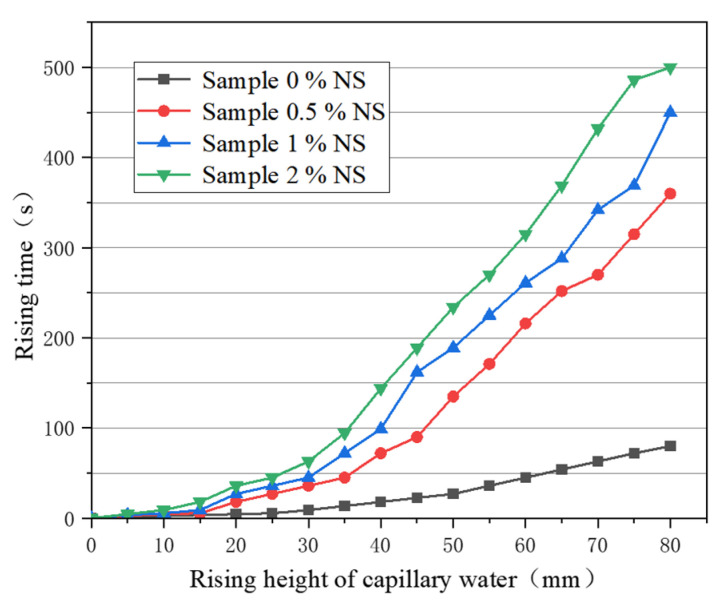
Relationship between rising height and rising time of capillary water.

According to the different dry mass ratios of nanosilica to aeolian dry powder soil, the proportion of nanosilica is 0, 0.5%, 1%, and 2% respectively. The test results are shown in Figure 4. It can be concluded that the rising speed of capillary water in the sample is different, the rising speed of capillary water in the sample 0% NS is the fastest, and it takes 80 s for capillary water to rise to the top of the sample. It takes 360 s for capillary water to rise to the top of sample 0.5% NS, which is 4.5 times as long as it takes the capillary water to rise to the top of the plain soil sample. It takes 450 s for capillary water to rise to the top of the sample with 1% NS, which is 5.6 times as long as for the plain soil sample. The rising speed of capillary water in the 2% NS sample is the slowest, taking 500 s to reach the top of the sample, 6.3 times as long as the plain soil sample. The main reason for the increase in rising time is that after nanosilica is added to silt, it disperses evenly in silt matrix, filling the pores of soil particles and increasing the density and strength of soil; thus, the rising speed of capillary water in the modified samples decreases and the rising time increases.

In summary, the capillary water effect can be effectively inhibited by adding nanosilica-modified materials to soil. The rising speed of capillary water in the sample decreases with the increase in nanosilica content, and the strength of soil can be improved by adding nanosilica modified materials.

### 3.2. Experimental Study on Combined Modification with Nanosilica and Potassium Methylsilicate

#### 3.2.1. Direct Shear Testing

**Figure 5:**
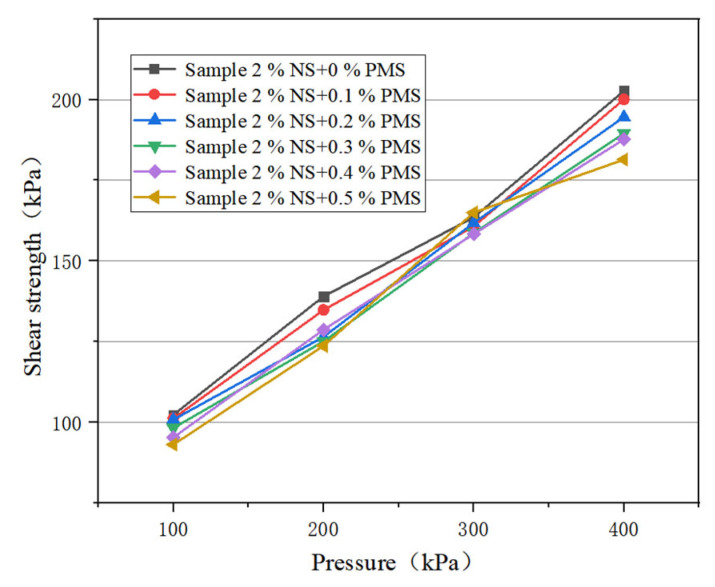
Shear strength curve of combined modified soil.

In order to study the influence of different amounts of potassium methylsilicate on the shear capacity of nanosilica-modified soil, 0, 0.1%, 0.2%, 0.3%, 0.4%, and 0.5% potassium methylsilicate solutions were added to the modified soil with 2% nanosilica content. The curve of shear strength change is shown in Figure 5. It can be seen that the shear strength of the samples decreases when 0.1%, 0.2%, 0.3%, 0.4%, and 0.5% potassium methylsilicate solution is added to the nanosilica-modified soil. Under the same vertical pressure, the shear strength of the sample decreases with the increase in potassium methylsilicate content. Under vertical pressure of 100 kPa, the shear strength of the samples with 0.1%, 0.2%, 0.3%, 0.4%, and 0.5% potassium methylsilicate is 101.2 kPa, 100.8 kPa, 98.2 kPa, 95.3 kPa, and 93.1 kPa, respectively, which is 102.3 higher than that of the nanosilica-modified samples without potassium methylsilicate solution.

**Figure 6:**
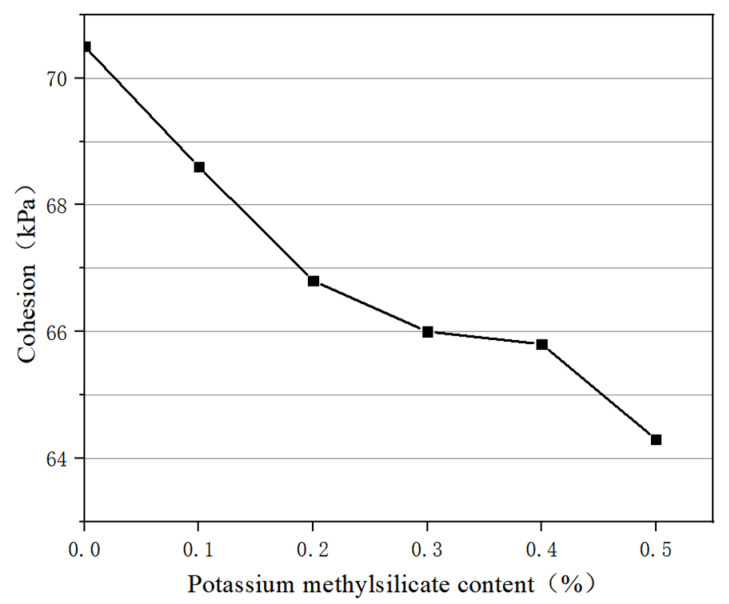
Variation of cohesion with potassium methylsilicate content.

The cohesion and internal friction angle of the modified soil sample were calculated according to the shear strength curve, and the graph of cohesion and potassium methylsilicate content was drawn as shown in Figure 6. It can be seen that the cohesion of nanosilica modified soil decreases with the increase in potassium methylsilicate solution content. When the dosage of potassium methylsilicate solution is 0.1%, 0.2%, 0.3%, 0.4%, and 0.5%, the cohesion of the soil modified by potassium methylsilicate and nano silica is 68.6 kPa, 66.8 kPa, 66 kPa, 65.8 kPa, and 64.3 kPa, respectively, which is 70.5 kPa higher than that of the soil modified by nanosilica without potassium methylsilicate solution.

**Figure 7:**
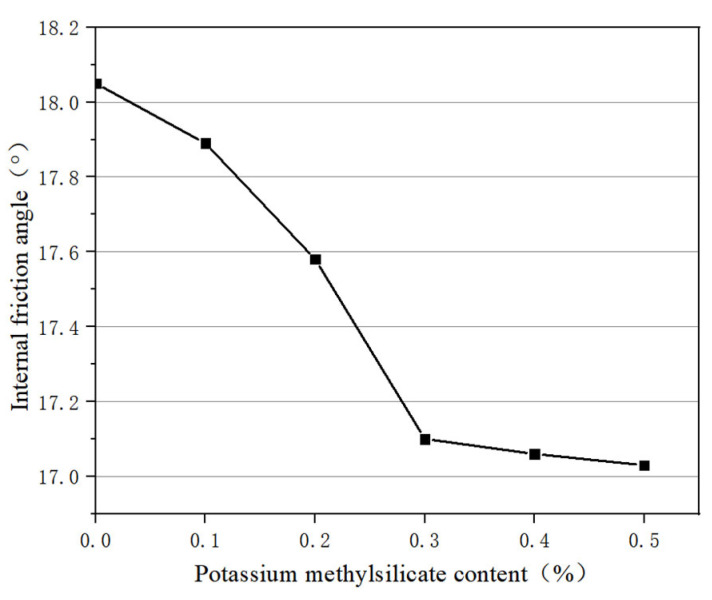
Variation of internal friction angle with potassium methylsilicate content.

The curve of internal friction angle and potassium methylsilicate content is shown in Figure 7. It can be seen that the internal friction angle of nanosilica-modified soil decreases with the increase in potassium methylsilicate solution content. When the content of potassium methylsilicate solution is 0.1%, 0.2%, 0.3%, 0.4%, and 0.5%, the internal friction angles of the soil modified by potassium methylsilicate and nanosilica are 17.89, 17.58, 17.10, 17.06, and 17.03, respectively; these values are higher than those of nanosilica without potassium methylsilicate solution.

The main reasons for the decrease of cohesion and internal friction angle of the sample are that nanosilica forms a gel when it meets water in silt, which fills the pores between soil particles, makes the soil more dense, and improves the shear strength. However, potassium methylsilicate has a slight retarding effect, reducing the mechanical properties that lead to gel formation of nanosilica when it meets water. Compared to nanosilica modified soil without potassium methylsilicate, the increase in soil compactness is reduced, thereby reducing the shear strength, cohesion, and internal friction angle of soil samples jointly modified by potassium methylsilicate and nanosilica.

In summary, the shear strength, cohesion, and internal friction angle of nanosilica-modified soil decrease with the increase in potassium methylsilicate solution.

#### 3.2.2. Capillary Water Rise Testing

**Figure 8:**
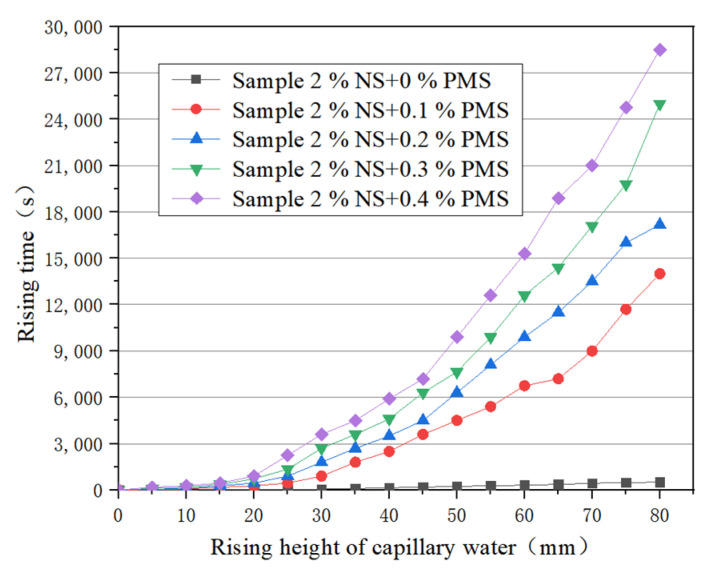
Relationship between rise height and rise time of capillary water in combined modified soil.

0, 0.1%, 0.2%, 0.3%, 0.4%, and 0.5% potassium methylsilicate solution was added to the modified soil with 2% nanosilica content. The relationship between the rise height and rise time of capillary water is shown in Figure 8. It can be seen that the rising speed of capillary water in sample (2% NS + 0% PMS) is the fastest, taking 500 s for capillary water to rise to the top. It takes 42 min for capillary water to rise to half the total height of the sample in sample (2% NS + 0.1% PMS), and 3.9 h hours for capillary water to rise to the top of the sample. It takes 58 min for capillary water to rise to half the total height of the sample in sample (2% NS + 0.2% PMS), and 4.8 h for capillary water to rise to the top of the sample. It takes 77 min for capillary water to rise to half the total height of the sample in sample (2% NS + 0.3% PMS) and 6.9 h for capillary water to rise to the top of the sample. It takes 98 min for capillary water to rise to half the total height of the sample in sample (2% NS + 0.4% PMS) and 7.9 h for capillary water to rise to the top. Capillary water hardly rises in sample (2% NS + 0.5% PMS), which shows that when 0.5% potassium methylsilicate solution is added to the nanosilica modified soil the capillary water essentially does not rise.

In summary, the rising speed of capillary water is faster in the nanosilica modified soil without adding potassium methylsilicate. After adding potassium methylsilicate to the nanosilica-modified soil, the rising speed of capillary water in the sample decreases with the increase of potassium methylsilicate content. When adding 0.5% potassium methylsilicate to the nanosilica-modified soil, capillary water essentially does not rise inside the sample. Thus, potassium methylsilicate effectively improves the waterproof performance of nanosilica modified soil.

### 3.3. Micro-Mechanism of the Composite Modified Soil

#### 3.3.1. SEM Testing Results and Analysis

**Figure 9:**
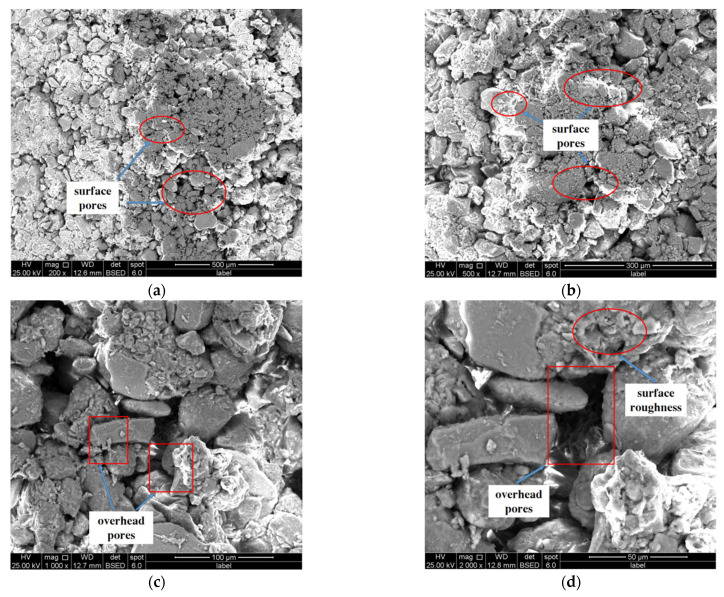
Microscopic image of plain soil under different multiples. (**a**) 200 times; (**b**) 500 times; (**c**) 1000 times; (**d**) 2000 times.

**Figure 10:**
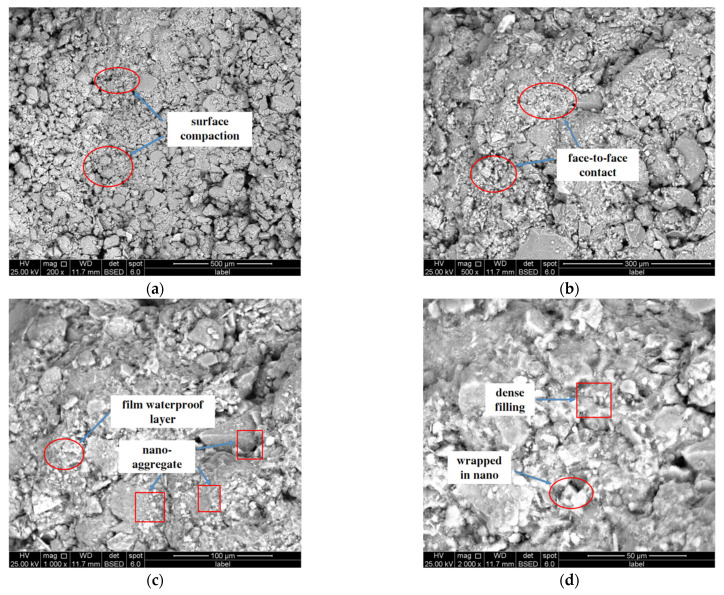
Microscopic image of combined modified soil under different multiples. (**a**) 200 times; (**b**) 500 times; (**c**) 1000 times; (**d**) 2000 times.

The SEM analysis results of the samples are shown in Figure 9 and Figure 10. Figure 9 shows the microscopic morphology diagram of each multiple of plain soil under electron microscope scanning. It can be seen that the sample soil particles of plain soil have different sizes and the surfaces of the particles are rough. The contact modes between soil particles are mainly point-to-point and point-to-face contact. The pores in the soil are large in number and shape, and there is little or no filler in the pores; overall, the whole soil structure appears very loose.

Figure 10 is the SEM image of the sample modified by 2% nanosilica and 0.5% potassium methylsilicate. Compared with the SEM microstructure of plain soil, the surface of the soil modified by nanosilica and potassium methylsilicate is very flat, the contact mode between particles is mainly surface contact, and the contact area is large. Furthermore, the pore size is relatively reduced, the number of pores is relatively reduced, the surface properties of soil particles have not changed, and there are a lot of fillers in the pores of soil particles, which makes the surface structure of soil more compact. Nanosilica can be evenly dispersed in the soil matrix, absorbing a small amount of water to form a gel state, filling the pores in silt aggregates, and improving the compactness of soil. In addition, nanosilica aggregates adhere to the surface of soil particles and expand from the particle surface to the particle edge, which increases the contact between soil particles and increases the particle size, thereby improving the mechanical properties of modified soil. A dense film is formed on the surface of soil particles. This is because when the potassium methylsilicate solution penetrates into the soil it reacts with water and carbon dioxide to decompose into methylsilicic acid and a polymethylsiloxane film is quickly formed to cover the surface of soil particles, forming a waterproof film on the surface.

#### 3.3.2. XRD Testing Results and Analysis

**Figure 11:**
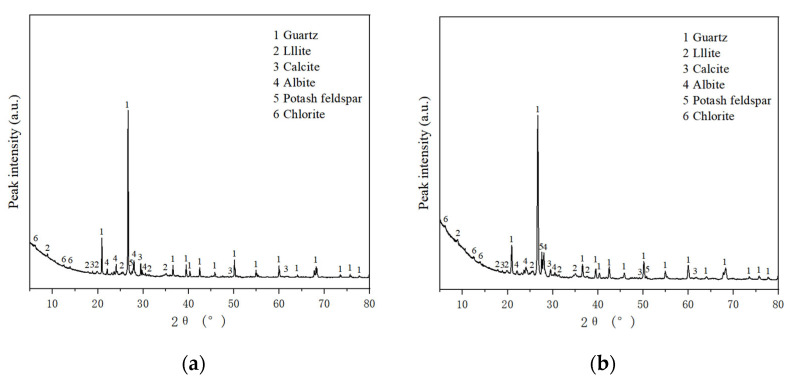
XRD testing results: (**a**) pattern of plain soil sample and (**b**) combined pattern of modified samples.

Figure 11 shows the XRD diffraction patterns of the plain soil sample and combined modified samples. Muñoz and V. Letelier et al. [26] detected similar mineralogical compositions using XRD in fired bricks made by partially adding bottom ash.

The XRD diffraction pattern of the plain soil is shown in Figure 11a. It can be seen from the peak curve in the figure that the main substances in the plain soil before adding modified materials are Quartz, Illite, Calcite, Albite, Potash feldspar, and Chlorite.

The XRD diffraction pattern of the soil modified by 2% nanosilica and 0.5% potassium methylsilicate is shown in Figure 11b. From the peak curve in the figure, it can be seen that the combined XRD diffraction patterns of the samples modified by nanosilica and potassium methylsilicate are essentially the same as those of the samples of plain soil; the quartz with higher peak values comes from the soil itself, while the illite, calcite, albite, potash feldspar, and chlorite contents of the soil do not change obviously with the addition of modified materials. It can be seen that the incorporation of nanosilica and potassium methylsilicate does not obviously affect the composition of soil or produce new substances. Nanosilica-modification of silty soil involves a completely physical mechanism, with physical changes such as filling, cementation, and wrapping occurring when it is added to silty soil. When nanosilica is added to silty soil, it forms a gel that fills the pores of soil particles, making the soil structure more dense. The improved strength of the resulting modified soil is can be observed macroscopically.

## 4. Conclusions

In this paper, potassium methylsilicate solution was added to nanosilica-modified soil and the composite modified soil was studied by mechanical property testing and waterproofing performance tests. The optimal ratio was then determined according to the relevant test results. The mechanism of joint soil modification via nanosilica and potassium methylsilicate was investigated from the microscopic perspective through SEM, EDS, and XRD testing, which can provide a theoretical basis for the protection and restoration of silty sites in Luoyang area. The relevant conclusions of this study are as follows:

(1) With increasing of nanosilica content, the shear strength of modified soil tends to increase; the cohesive force of 3% nanosilica modified soil is the largest, and is 43.8 kPa higher than plain soil. However, there is little difference between 3% nanosilica and 2% nanosilica, and higher nanosilica content can aggravate the brittleness of soil, leading to increased project costs. Therefore, it is suggested that the dosage of nanosilica be 2%. Adding nanosilica effectively reduced the rising speed of capillary water in the sample. The rising speed of capillary water in the 2% nanosilica-modified sample was slow, and took 500 s to rise to the top of the sample, which was 6.3 times that of the plain soil sample.

(2) With the addition of potassium methylsilicate solution, the shear strength, cohesion, and internal friction angle of the sample decreased. When the dosage of potassium methylsilicate solution was 0.5%, the cohesion and internal friction angle decreased by 8.79% and 5.65%, respectively. Moreover, with a potassium methylsilicate content of 0.5%, the phenomenon of capillary water rising hardly occurred in the sample.

(3) Through SEM and XRD tests, nanosilica was found to be evenly dispersed in the soil matrix; nanosilica absorbs a small amount of water to form a gel state that fills the pores in silt aggregates, thereby improving the compactness of soil. In addition, nanosilica aggregates adhere to the surface of soil particles and expand from the particle surface to the particle edge; this increases the contact between soil particles and increases the particle size, thereby improving the mechanical properties of the modified soil. When potassium methylsilicate solution is mixed into the soil, it reacts with water and carbon dioxide to decompose into methylsilicic acid and quickly generates a polymethylsiloxane film to cover the surface of soil particles, forming a waterproof film on the surface that improves the waterproof performance of the modified soil.

In this research, we only studied the effects of modified soil through laboratory tests; as the nanosilicon dioxide and potassium methylsilicate composite modified soil has not yet been applied in actual restoration engineering of earthen ruins, the real-world restoration effects of the composite modified soil have yet to be studied. Therefore, the next step involves field tests in which composite modified soil is applied to actual engineering repair in order to explore the composite modified soil repair process and its actual protection effect. In addition, follow-up work should be considered in order to add more field simulations of mechanical properties and waterproof performance testing in order to provide more specific references for the reinforcement and protection of earthen ruins.

## Figures and Tables

**Table 1:** Basic parameters of soil samples.

Property	Value
Sand (0.074~2 mm)	17.78%
Silt (0.002~0.074 mm)	82.11%
Clay (<0.002 mm)	0.11%
Liquid limit	25.1%
Plastic limit	16.3%
Plasticity index	8.7%
Maximum dry density	1.711 g·cm^−3^
Optimum moisture content	12.41%

**Table 2:** Particle composition of site soil.

Test Method	Particle Size Interval Group/mm	Less than a Particle Size Mass Fraction/%
Sieving method	>99.8	100
	2.0–1.0	99.15
	1.0–0.5	98.75
	0.5–0.25	98.55
	0.25–0.15	97.25
	0.15–0.075	90.30
Densimeter method	0.075–0.005	82.88
	0.005–0.002	5.05
	<0.002	0.13

**Table 3:** Parameter table of nanosilica.

Average Grain Diameter/nm	Pureness/%	Proportion/(m^2^/g)	Bulk Density/(g/cm^3^)	Colour	Form
20	≥99.8	200	0.05	White	Spherical granular powder

## Data Availability

Not applicable.

## References

[1] Bai Y. (2017). Research on the Application of Photoscan Multi-Angle 3D Reconstruction Technology in the Protection of Earth Sites. Master’s Thesis.

[2] Sun M. (2007). Present situation and progress of research on the protection of earthen sites. Sci. Conserv. Archaeol..

[3] Lv J., Liu X., Du Q. (2015). Research progress and engineering application of repairing materials for earthen sites. Relics Museol..

[4] Li Z., Zhang H., Wang X. (1995). Study on the Reinforcement of Ancient Earth Architecture Sites. Dunhuang Res..

[5] Li Z., Wang X. (1997). New progress in research on protection and reinforcement of ancient earthen architecture sites. Dunhuang Res..

[6] Wang X., Zhang L., Li Z., Wang C., Li W. (2002). Study on the present situation, protection and reinforcement of No.3 Mausoleum in Xixia, Yinchuan. Dunhuang Res..

[7] Zhou H., Zhang B., Chen G., Zhao H., Zeng Y., Guo Q., Li Z., Wang X. (2008). Study on in-situ protection and reinforcement of ancient earth sites in humid environment. Rock Soil Mech..

[8] Zhao H., Li Z., Han W., Wang N. (2008). Experimental study on strengthening earthen sites in arid areas of northwest China with PS materials. J. Hunan Univ. Sci. Technol. (Nat. Sci. Ed.).

[9] Bahmani S.H., Huat B.B., Asadi A., Farzadnia N. (2014). Stabilization of residual soil using SiO_2_ nanoparticles and cement. Constr. Build. Mater..

[10] Changizi F., Haddad A. (2015). Strength properties of soft clay treated with mixture of nano silica and recycled polyester fiber. J. Rock Mech. Geotech. Eng..

[11] Ren X. (2016). Experimental Study on Mechanical Properties of Frozen (Melted) Soil Improved by Nano Silica. Ph.D. Thesis.

[12] Ghasabkolaei N., Janalizadeh A., Jahanshahi M., Roshan N., Ghasemi S.E. (2016). Physical and geotechnical properties of cement-treated clayey soil using silica nanoparticles: An experimental study. Eur. Phys. J. Plus.

[13] Qu J., Hu C., Zhao C. (2017). Experimental study on shear strength of Shanghai clay reinforced by basalt fiber and nano silica. J. Water Resour. Water Eng..

[14] Shang Y., Fu Y. (2018). Experimental study of the mechanical properties of expansive soil with added nanomaterials. Arab. J. Geosci..

[15] Lv Q., Chang C., Zhao B., Ma B. (2018). Loess soil stabilization by means of SiO_2_ nanoparticles. Soil Mech. Found. Eng..

[16] Tomar A., Sharma T., Singh S. (2020). Strength properties and durability of clay soil treated with mixture of nano silica and polypropylene fiber. Mater. Today Proc..

[17] Tian Z., Lv X., Qin Y., He Y., Cui X. (2021). Preparation of PMS modified silica sol and its application in inorganic coatings. Mod. Chem. Ind..

[18] Lv X., Qin Y., Liang H., Liang H., Han Y.-C. (2022). Potassium methyl silicate (CH_5_SiO_3_Na) assisted activation and modification of alkali-activated-slag-based drying powder coating for protecting cement concrete. Constr. Build. Mater..

[19] (2010). Standard Test Methods for Liquid Limit, Plastic Limit, and Plasticity Index of Soils.

[20] (2019). Geotechnical Test Method Standard.

[21] Ahmed A. (2013). Recycled bassanite for enhancing the stability of poor subgrades clay soil in road construction projects. Constr. Build. Mater..

[22] Ahmed A., Ugai K., Kamei T. (2011). Investigation of recycled gypsum in conjunction with waste plastic trays for ground improvement. Constr. Build. Mater..

[23] Ju Z. (2021). Study on Shear Strength and Disintegration Characteristics of Loess Improved by Different Materials. Master’s Thesis.

[24] Kong R. (2019). Study on Mechanical Properties and Structural Characteristics of Loess Solidified by Nano Silica. Ph.D. Thesis.

[25] Jin Z. (2019). Experimental Study on Mechanical Properties of Soil Strengthened with Carbon Fiber and Nano Silica. Master’s Thesis.

[26] Muñoz P., Letelier V., Muñoz L., Gencel O., Sutcu M., Vasic M. (2023). Assessing technological properties and environmental impact of fired bricks made by partially adding bottom ash from an industrial approach. Constr. Build. Mater..

